# Axillary node status in breast cancer patients prior to surgery by imaging with Tc-99m humanised anti-PEM monoclonal antibody, hHMFG1

**DOI:** 10.1038/sj.bjc.6600200

**Published:** 2002-03-18

**Authors:** A R Al-Yasi, M J Carroll, D Ellison, M Granowska, S J Mather, C A Wells, R Carpenter, K E Britton

**Affiliations:** Department of Nuclear Medicine, St Bartholomew's Hospital, London EC1A 7BE, UK; Department of Surgery, St Bartholomew's Hospital, London EC1A 7BE, UK; Department of Histopathology, St Bartholomew's Hospital, London EC1A 7BE, UK; Imperial Cancer Research Fund, ICRF, Nuclear Medicine Group, London, UK

**Keywords:** breast cancer, axillary node status, Tc-99m hHMFG1, radiolabelled humanised monoclonal antibody imaging, radiolabelled anti-PEM, change detection analysis, probability mapping

## Abstract

In early breast cancer axillary nodes are usually impalpable and over 50% of such patients may have an axillary clearance when no nodes are involved. This work identifies axillary node status by imaging with a Tc-99m radiolabelled anti-Polymorphic Epithelial Mucin, humanised monoclonal antibody (human milk fat globule 1), prior to surgery in 30 patients. Change detection analysis of image data with probability mapping is undertaken. A specificity of 93% and positive predictive value of 92% (both 100% if a second cancer in the axilla with negative nodes is considered) were found. A strategy for combining negative imaging with the sentinel node procedure is presented.

*British Journal of Cancer* (2002) **86**, 870–878. DOI: 10.1038/sj/bjc/6600200 www.bjcancer.com

© 2002 Cancer Research UK

## 

When breast cancer presents early or when it is demonstrated by screening mammography, axillary nodes are often impalpable, so their involvement or not with cancer cannot be identified clinically. The need to determine axillary node status of patients with small cancers of the breast is evident for staging, management and prognosis. But over 50% of such patients have an ‘unnecessary’ axillary clearance at surgery in that the axillary specimen shows no involved nodes. The morbidity due to axillary clearance is acknowledged.

Biassoni *et al* (1998) tackled the problem of identifying tumour when axillary nodes were impalpable using Tc-99m SM3 monoclonal antibody imaging with success. F-18 DG deoxyglucose, positron emission tomography, PET, has also been used, but found unreliable in impalpable nodes (Wahl *et al*, 1991; Avril *et al*, 1996; Bombardieri *et al*, 1996; Utech *et al*, 1996; Crippa *et al*, 1998). The sentinel node approach is becoming the established technique for predicting axillary node status (Veronesi *et al*, 1997; Krag *et al*, 1998; Schlag and Veronesi, 2000). However the event is per-operative. The colloid used is not specific for cancer, only for identifying the node or nodes. Their excision has to be combined with histology. Imaging to determine axillary node status prior to surgery would allow the patient and the surgeon in principle to be prepared for axillary clearance as well as wide excision of the breast tumour or else wide excision alone. This study progresses the work of Biassoni *et al* (1998) by using a Tc-99m radiolabelled anti-Polymorphic Epithelial Mucin (PEM) humanised monoclonal antibody (human milk fat globule 1), hHMFG1. A simple strategy for combining this imaging with the current sentinel node approach is presented.

## MATERIALS AND METHODS

### Preparation of humanised hHMFG1 monoclonal antibody

Verhoeyen *et al* (1993) developed the anti-Polymorphic Epithelial Mucin, PEM, humanised monoclonal antibody (human milk fat globule 1), hHMFG1 by genetic engineering (Jones *et al*, 1986; Riechmann *et al*, 1988; Gorman *et al*, 1991). The master cell culture was shown to be virus free when used for the production of hHMFG1. This was purified by epitope affinity chromatography and immunoreactivity checked using purified milk mucin and fast protein liquid chromatography. Protein electrophoresis of the purified samples of the hHMFG1 antibody confirmed the presence of the heavy and light chains. The immunoreactivity of the humanised HMFG1 was found to be similar to the murine of over 90%. The relative affinities of the two antibodies were shown to be almost identical.

Epitope mapping was undertaken using the known epitope sequence synthesised as a series of amino acid sequences with the central PDTRP amino acid sequence. Removal of any one of the P, D, T or R led to loss of the binding capacity of either the human or the murine antibody indicating their specificity for the same epitope. From this data Verhoeyen *et al* (1993) concluded that the murine and the human reshaped HMFG1 are similar if not identical in their fine specificity and relative affinities for epithelial mucins. The preparation is aliquotted into vials in 1 mg amounts and transferred to the Nuclear Medicine Research Laboratory at St Bartholomew's Hospital. These are deep frozen at −80°C and used as required.

### Radiolabelling of hHMFG1 by photoreduction

Photoreduction of the antibody was performed using a standard laboratory transilluminator (UVP Inc, San Gabriel, CA, USA) containing two 300 nM UV tubes. Five ml of 50 mM PBS pH 7.0 containing 0.5 mM ethylenediaminetetraacetic acid (EDTA) was injected into a 10 ml nitrogen-filled borosilicate glass vial with a rubber septum and the solution was purged with nitrogen for 15 min. Five hundred μl of 5 mg ml^−1^ hHMFG1 antibody in PBS was injected into another 10 ml vial and the vial was purged with nitrogen for 15 min. An Amerscan Medronate II kit containing 5 mg sodium medronate, 0.34 mg stannous fluoride and 2 mg sodium p-aminobenzoate (Nycomed Amersham, Amersham, UK) was reconstituted with 2.0 ml of the nitrogen-purged PBS/EDTA. Two hundred μl of this medronate solution was injected into the vial containing the antibody and the vial was irradiated for 40 min 2 cm away from the exposed UV tubes of the transilluminator. The irradiated antibody was then divided into 500 μg aliquots in nitrogen-purged 2 ml vials and frozen at –40°C until required.

Photoreduced antibody prepared using this procedure was radiolabelled by addition to 500 μg of antibody of 740 MBq of sodium [99m-Tc] pertechnetate eluted from a 99-Mo/99m-Tc radionuclide generator (Nycomed Amersham, Amersham, UK) and incubation at room temperature for 30 min. Radiochemical purity of the labelled preparations was measured by HPLC and ITLC methods as previously described (Stalteri and Mather, 1996) and in all instances exceeded 95%. Immunoreactivity of the photoreduced antibody was compared with the native antibody using a solid-phase ELISA assay on plates coated with a GST-MUC1 fusion protein (Apostolopoulos *et al*, 1993; Wilkinson *et al*, 2000). No significant differences were seen between the binding of the reduced and control antibody preparations.

### Patients

All the patients were referred from the surgical breast clinic, mostly from the breast screening programme. The breast clinic is run twice weekly at St Bartholomew's Hospital. It is a ‘One stop’ clinic, where radiology and cytology are available directly after the patient has been seen. In this clinic a full medical history is taken with emphasis on the breast problem and any family history of breast cancer, followed by a careful examination of breasts. Both axillae and supraclavicular regions are examined for any palpable nodes and the findings are recorded.

The patient then has mammography of both breasts. Ultrasound is routinely done for the breasts and to the liver. All the patients have a fine needle aspiration to examine the cytology of the breast lump at the time of their attendance or afterwards. Some of these patients have to have a core needle biopsy to confirm the suspicion of non-diagnostic fine needle aspiration or non-diagnostic radiological findings.

Once the treatment options have been explained, the patient has the radiolabelled monoclonal antibody imaging explained and informed signed consent is obtained. The patient is asked about any allergies to inoculations. If positive the patient would be excluded from the test but no such patient was found so none were excluded. The breast surgery is usually undertaken within a month of the histological diagnosis and the imaging with Tc-99m hHMFG1 within 2 weeks before surgery.

### Preparation of the patient for imaging

The patient is asked to wear the same bra for each study. The activity to be injected is checked. Six Cobalt-57 markers are placed over bony and other landmarks, previously marked with an indelible marker. These are the reference points for next day imaging. The Co-57 landmarks are positioned on the suprasternal notch, bilateral mid-clavicular line, xiphisternum, the nipple of the diseased breast and the tumour site. In case of right breast tumour, a flexicurve lead is used to outline the breast to identify it separately from the liver. For the left breast no flexicurve is used.

The usual activity is 600 MBq of Technetium-99m labelled to 0.5 mg hHMFG1, which is injected intravenously when the patient is lying down supine on the camera couch. In the case of bilateral breast tumours the injection is given in the arm opposite to the side of the planned axillary clearance. The patient is positioned supine with the arms at right angles to the body on armrests. The camera is positioned anteriorly over the chest. By the help of a transparent X-ray film attached to the computer screen, the six markers are marked on the film, using a permanent ink pen.

This transparent film is used as a reference for imaging the patient at 3 h and 24 h. The transparent film is cut to the size of the computer screen and fixed on the surface of the screen using tape. All six markers are marked on the film specifying the tumour and the nipple sites. Then the images are acquired. Two sets of static anterior chest images are obtained on the first day: 10 min post injection and at about 3 h post injection. A further image is obtained the next day at about 24 h. The Orbiter gamma camera (Siemens, Erlangen) linked to Hermes computer system (Nuclear Diagnostics, Stockholm) is used. The gamma camera is peaked on an energy level of 140 KeV, window 15%, set with a matrix size of 256×256 and with a general purpose high resolution collimator.

Ten minutes post injection, the six markers' view is acquired for 30 s. Then 1000 K counts are acquired for an image of the anterior chest with the view including both breasts and axillae extending from the cricoid cartilage to costal margins. The image data is transferred to the computer system. Three to 4 h and 18–24 h post injection, the procedure is repeated. The planar images are reviewed and the image data undergoes Change Detection Analysis, CDA.

## Interpretation of images

The planar images are reviewed. The change detection analyses are presented as probability maps of the significances of the differences of the images at pairs of time points. These are colour coded: Red is *P*<0.001; Yellow, *P*<0.01; Green, *P*<0.05; Blue; *P*⩾0.05, Dark blue or Black, *P*>0.1 for the significance of the differences. *P*<0.05 is taken as the divide between a positive and a negative detection of axillary node involvement. Findings of the probability mapping were evaluated to take into account other areas with increase of uptake. These may be due to imperfect registration or repositioning of the patient during the 22 h image. Areas above the brachial and subclavian arteries where there was with CDA an increase with time of radiolabelled antibody were considered not relevant, because of frequent artefacts due to neck movements. Small cancers do not usually spread above the clavicle.

As the liver is the organ where antibodies are metabolised, there was always an increase of activity with time, so the organ was isolated in a region of interest and excluded. Areas of negative change including the heart and big vessels were also isolated in region of interests and excluded.

For hHMFG1 imaging, the planar images are called negative or positive on visual inspection of the black and white images prior to knowledge of the CDA findings. The outcome of Tc-99m hHMFG1 imaging of the axilla is reported as TP true positive, FN false negative, TN true negative and FP false positive in relation to the histological findings.

### Specimen handling

The Pathology Department receives the pathology form on which the patient data and clinical findings are recorded, together with labelled specimens. The written record of the activity counts is also provided. All specimens are placed on the tray designated for use only for radioactive specimens. The pathologist monitors the specimens by using a Geiger–Muller counter, recording the counts of each specimen separately. The pathologist dissects and separates the lymph nodes from the axillary clearance specimens. The pathologist also separates the sentinel node from its bed when that technique has been performed, and dissects the primary tumour. The specimens are stored in formalin solution in separate containers and kept in a lead box in the pathology laboratory until ready for processing. The nodes have multiple sections depending on their size. If nodes are macroscopically positive then one section only is taken, otherwise nodes greater than 5 mm have multiple sections, those less than 5 mm are embedded in their entity. The breast tumour is examined routinely. Haematoxylin and eosin staining is performed for each of the sections taken from each block. Immunocytochemistry is not used routinely. The grading is performed by the Elston and Ellis method, dividing tumours into grade 1, 2 and 3, as well as a short description of the type of breast cancer e.g. invasive ductal carcinoma. DCIS is ductal carcinoma *in situ* that is confined within the ductal system.

### Change detection analysis, CDA, with probability mapping

Lymph nodes are small, sometimes deep and malignant cells show relatively low antibody uptake, of the order of 0.01% injected dose per gram. This makes it difficult for conventional visual interpretation of planar images to be reliable and to confirm such nodes as being clinically involved. The detection of an involved lymph node is essentially that of detecting a faint radioactive signal in a background of radioactive noise. For such lesions a tumour to noise ratio or contrast, ct, can be defined as:


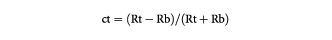


Where Rt is the counting rate recorded over the lesion and Rb is the counting rate recorded adjacent to it (Sorenson and Phelps, 1987).

The background activity also has an effect on the contrast ratio. Taking the background to exhibit a uniform counting rate of Ro, which is then added to the image, then the tumour contrast ratio is now given by:


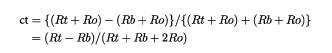


From this expression it may be seen that the tumour contrast ratio is reduced by the additional factor 2Ro in the denominator.

Observer studies have shown that the minimum tumour contrast ratio for tumour sites to be detected reliably by the human visual system, usually using transparent film, is at least five.

As the tumour to background ratio is usually low and as scattering and attenuation of photons within the patient is another problem, so statistical probability mapping, SPM, with a change detection algorithm has been investigated to see if it provides an acceptable solution.

The process of SPM is that of deriving a parametric image whose elements contain the significance *P*-value returned from performing some parametric or non-parametric statistical test between the pixels of the two images being compared. There may be considered to be two distinct approaches to SPM comprising: Global linear regression and pixel by pixel statistical comparison.

Global linear regression utilises a two-dimensional co-occurrence matrix or scatter graph. This matrix may for example have dimensions 256×256, where the matrix size determines the maximum count that can be processed in the images being compared. Each pixel count in the two images A and B being compared is used to index into the co-occurrence matrix where the pixel counts in image A is used as a column index and the respective pixel counts in image B provide the row index. Each such location in the co-occurrence matrix is incremented by one hence the result after processing all the pixel counts in images A and B is that of a two dimensional histogram describing the joint pixel count distribution between the two images. If the images are identical the results fall on the line of identity. Deviations from this line can be tested for significance using a Chi square test. A Cluster requirement can then be applied so that, for example, five pixels with significant deviations must be adjacent to be judged significant. This avoids single outlier pixel values. The probability map is then constructed.

The two dimensional co-occurrence matrix approach is a relatively simple approach to statistical change detection providing a straightforward statistical significance and sense i.e. positive or negative areas of change between successive homologous images. This approach has been used successfully in ovarian cancer (Granowska *et al*, 1988) but suffers from some potentially serious disadvantages.

These are firstly the assumption of a linear relationship between the counts in the two images being processed. This assumption may be violated by variations in gamma camera response between data acquisitions taken 24 h apart. Secondly and more serious is that large areas of very positive change, for example the liver in immunoscintigraphy, will dominate in calculating the regression line thus swamping the contributions from small areas of positive change with far fewer pixels.

Because of these limiting factors the second approach to SPM was investigated called Statistical Pixel by Pixel Comparison.

Localised pixel comparisons between images was used as the method of choice for the detection of axillary lymph node involvement, because of the disadvantages of global linear regression mentioned above. Prior to statistical pixel comparison an image normalisation or pre-whitening stage is carried out. Besides aiding in the detection process directly this image normalisation process has a second important consequence. When performing a statistical test upon a set of data then the power of any such test is directly dependent upon the assumption of independence on each element within the data set. But in the case of image data such independence assumptions are seen not to hold by virtue of each pixel being highly correlated with its neighbours. This interpixel correlation is a consequence of the imaging equipment's finite point spread function or resolution at the short scale, and, at larger scales from sampling common large underlying structures such as the liver in immunoscintigraphy. The image normalisation process ensures a significant pixel to pixel decorrelating effect and therefore the independence requirement of a chosen statistical test is largely met. This normalisation process takes the form of a transformation described by the expression:


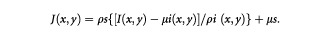


Where (μ*i*) and (*i*) are the local mean and standard deviation of the input image, (I), measured over a local (n×n) window centred at the current pixel position *x*,*y* and where (μ*s*) and (ρ*s*) are the required mean and standard deviation of the output image (J).

This mapping is called statistical scaling and is related to the image processing technique of unsharp masking. A window, 5×5 pixels in extent has been chosen for the current application. For the two homologous images, a small-centralised window or group of pixels is extracted for each pixel, using the window size of 5×5 pixels square. For each of these small sub-populations a parametric or nonparametric test statistic may be calculated for some property associated with the image pixels. This property is usually the radioactive counts per pixel but it is important to stress that any derived quantity from the images may be used as a valid input, for example measures of surface shape or texture. The chosen parametric statistical test for this application is that of the paired *t*-test where the significance of any difference in the mean counts of the windowed pixel subset is tested.

The null hypothesis that a significant difference or a tumour is not present (H0) is rejected when for (na+nb-2) degrees of freedom of the *t* distribution, the level of significance, *P*-value, returned by the test is greater than a prescribed level of significance e.g., a *P*-value >0.05.

With a 5×5 window size, na, nb=25 and where a and b are the means of the sample values, there are 25 + 25 −2=48 degrees of freedom. Referring to statistical tables of the t distribution, a value of ‘*t*’ greater or equal to 2.42 would indicate a *P*-value of 0.005, a highly significant result and the null hypothesis (H0) would be rejected. Various *P*-values are presented by mapping to some chosen colour table. For this application *P*<0.001 is plotted in red, *P*<0.01 in orange, *P*<0.05 in green, *P*⩾0.05 in blue and *P*>0.1 in dark blue or black.

In summary, the process of generating the SPM parametric image is image registration, change detection and image output as a probability map.

### Results of breast and axillary imaging

The 32 patient's ages ranged from 40–80 years old, mean 57.3 years, SD 10.7 years. The breast tumours ranged from 10–50 mm diameter with a mean of 23.6 mm, standard deviation 11.3 mm. There were 17 patients with cancer in the left breast and 15 in the right breast. The positions of the tumours were mainly upper outer quadrant of the breast, 22; upper inner quadrant, three; lower outer quadrant, six; lower inner quadrant, none; and one central tumour. The patient data and the correlation with histology are shown in Table 1

**Table 1:**
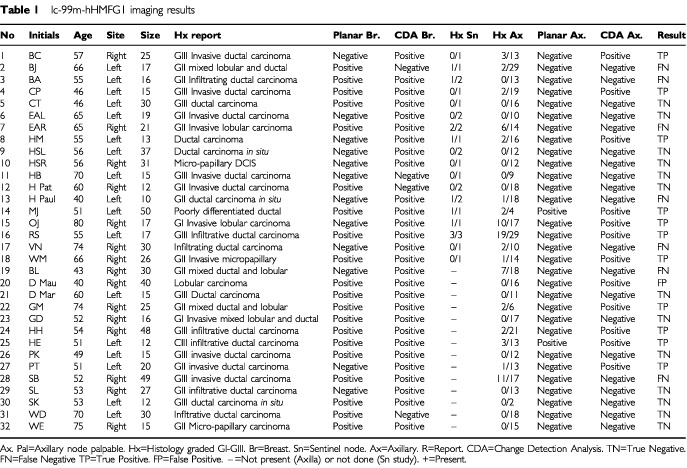
Ic-99m-hHMFG1 imaging results

. Of the total of 478 lymph nodes sampled in these 32 women, 87 showed histological involvement with breast cancer. A subset of 26 sentinel nodes in their axillae showed involvement in 11, Table 1. Both true positive and false negative images were found when only one or two out of many axillary nodes were involved, Table 1. The results in Table 2

**Table 2:**
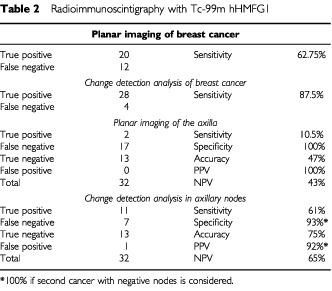
Radioimmunoscintigraphy with Tc-99m hHMFG1

show the advantage of change detection analysis over planar imaging. Representative images are shown in Figures 1

**Figure 1:**
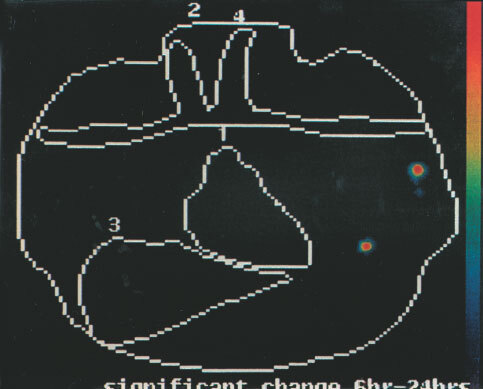
Patient CP 4, Tc-99m hHMFG-1 radioimmunoscintigraphy. Positive left axillary nodes and breast cancer.

, 2

**Figure 2:**
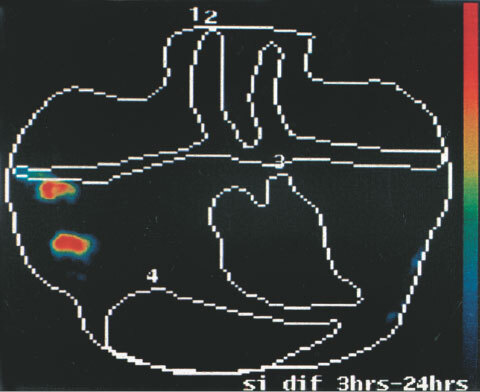
Patient BC 1, Tc-99m hHMFG-1 radioimmunoscintigraphy. Positive right axillary nodes and breast cancer.

, 3

**Figure 3:**
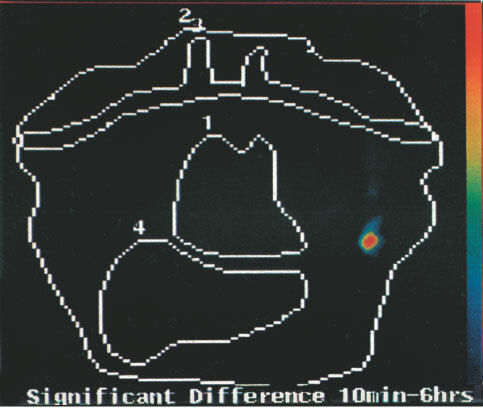
Patient CT 5, Tc-99m hHMFG-1 radioimmunoscintigraphy. Negative left axilla nodes and positive breast cancer.

, 4

**Figure 4:**
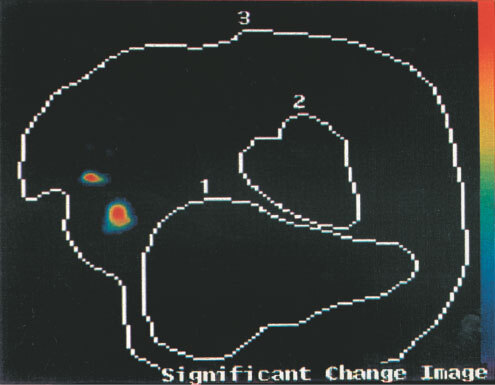
Patient W M 18, Tc-99m hHMFG-1 radioimmunoscintigraphy. Positive right axillary nodes and breast cancer.

.

The pharmacokinetics of Tc-99m hHMFG1 were investigated. The T1/2 slow component was approximately 26 h. The 24 h urine output of Tc-99m labelled metabolic products, usually Tc-99m dicysteine, was approximately 50% of the injected activity.

## DISCUSSION

Radioimmunoscintigraphy of breast cancer started in this department with murine monoclonal antibody 19 years ago (Epenetos *et al*, 1982). The aim of this study was to detect malignant involvement in impalpable axillary lymph nodes prior to surgery in women with proven breast cancer by imaging with a technetium-99m radiolabelled ‘humanised’ monoclonal antibody.

Axillary clearance, although associated with some morbidity, is considered an essential and standard part of an operation to remove breast cancer in order to stage the disease. Nodal status determines prognosis and the choice of therapy. However, up to 50% of these women with breast cancer over 1 cm diameter have an ‘unnecessary’ axillary clearance in that the nodes are shown histologically to be free of cancer. The reliable determination by imaging of involvement or not of impalpable axillary nodes in the days prior to surgery would allow the surgeon to tailor the extent of the operation to the individual woman, who would be forewarned. A positive image would indicate the need for this clearance and a normal image would indicate no need. However, it is difficult to prove the absence of a micrometastasis when an image of the axilla is negative. A strategy, in which there is the addition of the Sentinel node technique per-operatively, is intended to confirm a negative as true and thus confirm that axillary clearance would not be necessary.

Radiological techniques, X-ray CT, MRI and or ultrasound have a ‘normal’ cut off limit of 1 or even 1.5 cm diameter. Thus a node less than 1 cm diameter, which is detected by these techniques, has to be considered normal or else these techniques would have an unacceptable high false positive rate for cancer detection in lymph nodes. Yet it is clear that for a cancer to enlarge a node to over 1 cm diameter, it must have been present in a normal size node.

The success of radioimmunoscintigraphy in identifying small cancers, local involvement of normal sized lymph nodes and recurrent cancers that spread in sheets or ribbons or recur as plaques, is that the technique is essentially one of identification or characterisation of a tissue type. It is making use of the subtle differences between the cancer cell and the normal cell. A radioactive pinhead is identifiable if it has enough radioactivity on it, although it may appear over a centimetre across on the gamma camera image because of that imaging system's inherently poor resolution. It is the number of molecules that contain a radioactive signal that bind to each cancer cell that is the crucial determinant. Where tumour associated antigens are concerned there are between 5000 and 50 000 accessible antigens per cell. It has been calculated that there are up to 10 000 HMFG1 epitopes accessible for binding on a typical malignant breast cancer cell surface. This in principle gives a considerable amplification factor, which helps to overcome the poor photon yield of radiopharmaceuticals with gamma camera imaging. The order of magnitude greater detection sensitivity over single photon agents of PET equipment for Positron Emitting F-18 of FDG helps to account for its success. However the amplification of the Glucose Transporter protein 1 because of its upregulation in malignancy as compared to normal tissue appears to be only five to 10-fold.

The polymorphic epithelial mucin, PEM, antigens are epithelial antigens hidden from the blood stream in normal tissues such as the lining of the lactiferous duct in the breast or the internal surface of an ovarian follicle. It is the architectural disruption and disorganisation of the malignant process that exposes such antigens to the blood stream as well as their up regulation that allows cancer detection by monoclonal antibodies such as HMFG1 either immunohistochemically or by radioimmunoscintigraphy.

The detection system has to be optimised to obtain the best images. There is a further consideration. The time dependency of biological processes is one of nuclear medicine's greatest strengths (Britton and Granowska, 1987). This time dependency of monoclonal antibody uptake by cancer is the key to the further specificity of radioimmunoscintigraphy for detection of small lesions. Specific uptake increases with time. Thus an image at 5–10 min at which time there is virtually no uptake of the monoclonal antibody by the cancer, acts as a template with which to compare the later images. This is the basis of the change detection analysis, CDA, also called statistical probability mapping, SPM, which latter has been used for functional MRI of the brain, reviewed by Acton and Friston (1998). In contrast non-specific uptake after the initial distribution decreases with time as the agent diffuses out of the lesion as the blood level of the antibody falls with time.

To undertake CDA involves correct registration of the images. This is firstly by setting the patient in the same position for each view as far as possible by the use of the markers and the transparent film procedure. Secondly by the computer's translation rotation program. Superimposable images are a requirement for the change detection analysis program to be applied. This tests the significance of the difference between the two images, the early and late. This significance is plotted as a colour coded probability map. The sophisticated version of CDA used here followed the earlier simpler approach, published first in the context of ovarian cancer by Granowska *et al* (1988), and the more recent refined technique applied to the imaging of the axillary nodes in breast cancer using Tc-99m SM3 (Biassoni *et al*, 1998). It has been further refined using the 25 cell matrix whose central pixel is applied successively to each pixel of each image. This approach allows for some slight movement and distortion between the two images and gives a robust probability map.

The CDA has a number of problems. The registration is crucial. Alignment of the patient using the markers and transparent film is the first step, but it does not allow for twisting of the patient's body. For this reason, although theoretically only three markers are needed to define co-registration, six markers are used. The two for the site of the tumour and the nipple are less reproducible but aid the interpretation of the image. The marker images are then analysed to find the pixel with the maximum count rate for each marker. A direct transform is undertaken if the marker images for the early and later image fit, otherwise an elastic transform minimising the differences between the marker images has to be undertaken. The actual breast and axillary node images are transformed in the same way as their marker images. Errors in registration may be seen as negative shadows related to blood vessels, but the movement artefacts can be deceptive. All those on or above the line of the subclavian arteries and in the neck are ignored since they are typically due to head and neck movement of the patient between images. As the patients are mainly from the Breast screening programme and have small tumours, supraclavicular nodes are most unlikely to be involved. This technique is not able to detect them because of these movement artefacts. It is focussed on the need for defining axillary node involvement in relation to axillary clearance.

The results of radioimmunoscintigraphy with Tc-99m humanised hHMFG1 with change detection analysis show good specificity: 13 out of 14 true negatives. The one false positive, patient 20 was due to a second tumour found histologically to be breast cancer and not due to an involved lymph node. Although a relatively rare occurrence, a second tumour having the same antigen expression as an involved lymph node, cannot be distinguished by radioimmunoscintigraphy. The result is a true positive for cancer cell detection and probably a true positive in management terms, since if a tumour was known to be in the axilla, an axillary clearance would be undertaken. The sensitivity 61% however is disappointing with seven false negative studies in patients 2, 3, 7, 13, 17, 19 and 28. There are several likely explanations. There is lack of sensitivity of the technique in detecting what were micrometastases in patients 2, 3 and 17. The situation in patient 7 (two sentinel nodes and six out of 14 axillary nodes involved), in patient 19 (seven out of 18 axillary nodes involved) and in patient 28 (11 out of 17 axillary nodes involved) is likely to be due to a paucity of antigen expression in these tumours' metastases to the axillary nodes. In each case the primary tumour was detected. Patient 7 had a grade 2 invasive lobular carcinoma, patient 19 had a grade 2 mixed ductal and lobular carcinoma and patient 28 had a Grade 3 invasive ductal carcinoma. These are not more malignant than the average grade of the other true positives. These results of CDA with Tc-99m hHMFG1 are less good than those of a previous study from this department (Biassoni *et al*, 1998) using CDA with Tc-99m-SM3. SM3 has a higher affinity for breast cancer than murine HMFG1, whose affinity is similar to the humanised version of HMFG1. The comparison between this study and the SM3 study is historical, for the patients were not studied with each antibody. However the patients were similar in presentation with the same surgical team attending a similar number of patients. Unfortunately it has not been possible to humanise SM3 which reacts with a five amino acid epitope exposed through the relative deglycosylation of the cancer cell surface mucin protein as compared with the normal breast cell or benign breast lesion (Taylor-Papadimitriou *et al*, 1981; Burchell *et al*, 1987, Burchell and Taylor-Papadimitriou, 1993). This makes it more specific for cancer. The advantage of a humanised as compared to a murine monoclonal antibody is not primarily for imaging where the HAMA reaction is not a problem until the third or fourth diagnostic imaging is undertaken, but for the requirements of radioimmunotherapy or inoculation against cancer.

In conclusion the applicability of using a Tc-99m humanised anti-PEM monoclonal antibody in imaging impalpable axillary nodes in women with breast cancer has been demonstrated. The next phase is to return to the use of Tc-99m-SM3, which had a better sensitivity than hHMFG1, with the more advanced CDA image analysis technique described and used here.

A strategy for management of axillary clearance is proposed in Table 3

**Table 3:**
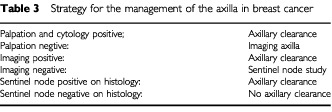
Strategy for the management of the axilla in breast cancer

. The imaging technique may be improved radioimmunoscintigraphy, improved radiopeptidescintigraphy, improved positron emission tomography, or improved magnetic resonance imaging. The combination of negative palpation of the axilla, negative imaging of the axilla and a negative sentinel node study would be taken as the criteria to avoid axillary clearance.
